# Supplementary material for: Does environmental management system certification keep enterprises out of trouble? Evidence from stock price crash risk
Source: PLoS One. 2024 Dec 30;19(12):e0311740. doi: 10.1371/journal.pone.0311740 (PMC11684633; doi:10.1371/journal.pone.0311740)
Supplement: Attachment — Submitted filename: Response to reviewers.docx [file pone.0311740.s002.docx]

Response to Reviewers

Dear Editor and Reviewers,

We appreciate the detailed and constructive feedback provided by the reviewers. We have carefully considered each comment and have made significant revisions to our manuscript to address the concerns raised. Below, we provide a point-by-point response to the comments from both reviewers.

Reviewer #1:

Comment 1: The study does not use anything new in terms of dependent variables, as they use the dated Chen et al. (2001) study variables DUPOL and NCSKEW (that the authors just rename in this study DUP and NCSK).

Response: We acknowledge the use of established variables from Chen et al. (2001). These variables, DUPOL and NCSKEW, are robust measures of idiosyncratic risk and have been widely used in similar studies. We have retained these variables for consistency and comparability with previous research. However, we have added new robustness tests using standard deviation and beta to measure volatility and systematic risk, respectively, as suggested. Additionally, we have explicitly cited Chen et al. (2001) in the revised manuscript from line 289 to 291.

Comment 2: The authors mislabel the measures as 'volatility', while in the original Chen (2001) study it is referred to as 'idiosyncratic risk'. The authors refer to these variables as first used by Wang et al. (2021; Duan and Lin, 2021), which is incorrect. Also, the reference to Wang (2021) is missing.

Response: We apologize for the oversight. We have corrected the terminology to 'idiosyncratic risk' as used in Chen (2001). We have also removed the incorrect reference to Wang (2021) and clarified that Duan and Lin (2021) are valid references. The source of equations 3 and 4 has been correctly attributed to Chen (2001) in the revised manuscript.

Comment 3: The introduction, literature review, and hypothesis tested sections are poorly written and lack proper referencing. They should be rewritten to include references to recent studies and to provide a coherent flow.

Response: We have completely rewritten the introduction to focus on the study's contribution in light of past literature and the context of China from line 30 to line 268. The literature review has been revised to include more relevant and recent references, emphasizing studies on idiosyncratic risk and ISO14001/EMS. Each hypothesis now connects the study to a major theory, and we have added multiple references to support our hypotheses. Specifically, references to Zaman (2021) and Mosgaard (2022) have been replaced with more relevant studies.

Comment 4: The Research Findings and Conclusion sections should be rewritten to clearly show the contribution of the study and connect findings to other research.

Response: We have revised the Research Findings and Conclusion sections to clearly delineate the contributions of our study from line 496 to line 549. These sections now explicitly connect our findings to existing research, highlighting the new insights provided by our study.

Reviewer #2:

Comment 1: Similarity with Previous Work: Please provide a detailed comparison between the current study and your previous work.

Response: I didn’t find the article you mentioned (Qiao, Liu, & Qin, 2023). In the previous study, we examined the influence of environmental management system (EMS) certification on stock price volatility and the risk of stock price collapse in the context of Chinese manufacturing firms. The key contributions of the current manuscript include:

Policy Impact Analysis: While the earlier work discussed general relationships, the current study specifically analyzes the effect of the new Environmental Protection Law implemented in 2015, showing that the association between EMS certification and reduced stock price collapse risk has become more significant post-implementation.

Sector-Specific Insights: This study expands the scope by focusing on heavily polluting enterprises and state-owned enterprises, highlighting the differential impact of EMS certification in these contexts.

Mediating Role of Media Coverage: A novel contribution is the detailed analysis of how media coverage influences the relationship between EMS certification and stock price collapse risk, adding depth to the understanding of informational mechanisms at play.

Comment 2: Differences Between EMS Certification and Environmental Regulation: Elaborate on how EMS certification differs from mandatory environmental regulations.

Response: We have included a detailed discussion on the differences between EMS certification and mandatory environmental regulations. This section elaborates on EMS certification as a voluntary and participatory mechanism, contrasting it with mandatory regulations, and explains its unique significance in influencing stock price crash risk, supported by references to Boiral (2007) and King, Lenox, & Terlaak (2005).

Comment 3: New Significance of Findings: Emphasize the new significance of your findings in the context of existing literature.

Response: We have highlighted the new significance of our findings in advancing the understanding of the economic consequences of EMS certification. This discussion emphasizes EMS certification's role as an environmental governance and information transfer tool, building on the works of González-Benito & González-Benito (2008) and Jiang (2020).

We hope that these revisions adequately address the reviewers' comments and improve the clarity and comprehensiveness of our manuscript. We thank the reviewers again for their valuable feedback and look forward to your response.

Sincerely,

Hongyu Liu
